# Magnitude of Psoriasis and Its Associated Factors, and Quality of Life of Psoriasis Patients among Patients Who Attend Dermatology Clinic at Tibebe Ghion Comprehensive Specialized and Addis Alem Primary Hospitals, North West Ethiopia, 2022: Institution-Based Cross-Sectional Study

**DOI:** 10.1155/2024/5560174

**Published:** 2024-09-18

**Authors:** Wosen Ketema, Solomon Ketema Bogale, Eyob Ketema Bogale

**Affiliations:** ^1^ School of Medicine and Health Sciences Department of Dermatovenereology Bahir Dar University, Bahir Dar, Ethiopia; ^2^ Department of Nutrition Antsokiya Gemza Wereda Health Office, North Shoa, Ethiopia; ^3^ Department of Health Promotion and Behavioral Sciences College of Medicine and Health Science Bahir Dar University, P.O. Box 79, Bahir Dar, Ethiopia

## Abstract

**Background:**

Psoriasis is a common immune-mediated papulosquamous inflammatory skin disease characterized by well-demarcated, erythematous silvery scaly plaques. Prevalence rates of psoriasis showed a worldwide variation and have been reported to range between 0.09% and 5.1%. It has been associated with several intrinsic as well as extrinsic factors and has a detrimental effect on health-related quality of life. Studies on the burden, factors associated with it, and quality of life of psoriasis are limited in the study area.

**Objectives:**

To assess the magnitude, factors associated with it, and quality of life of psoriasis patients.

**Methods:**

Institution-based cross-sectional study was conducted among patients who attend dermatology clinics at Tibebe Ghion comprehensive specialized and Addis Alem primary hospitals from June 8 to September 5, 2022. Systematic random sampling was applied to select study participants until the sample size (318) was fulfilled. A structured interviewer-administered questionnaire was used to collect data. Collected data were coded and entered into EPI data version 3.1 and then exported to SPSS version 27 for analysis. Descriptive statistics and logistic regression analysis were done.

**Result:**

The mean age of study participants was 22.5 with a standard deviation (SD) of 18.2 years. The proportion of psoriasis was 5.3% with a 95% CI (3.1%, 8.4%). Stressful life events (AOR = 3.32, 95% CI (1.12, 9.86)) and alcohol consumption (AOR = 3.73, 95% CI (1.03, 13.51)) were significantly associated with psoriasis. Seven (41.2%) psoriasis patients had a large effect on their quality of life. The mean dermatology quality of life index (DLQI) was 9.82.

**Conclusion:**

The proportion of psoriasis in this study was comparable to earlier international and Ethiopian studies, but greater than studies from other African countries. Stressful life events and alcohol consumption were significantly associated with psoriasis. The majority of psoriasis patients had a large negative effect on their quality of life.

## 1. Introduction

Psoriasis is a common immune-mediated, papulosquamous inflammatory skin disease. It is characterized by pruritic, well demarcated red, erythematous silvery scaly plaques occurring most commonly on the scalp, trunk, elbows, and knees, but any skin surface can be involved [1].

Although there are several clinical types of psoriasis, the plaque type, or psoriasis vulgaris, accounts for more than 85% of cases. Other uncommon types of psoriasis include sebopsoriasis, erythrodermic, pustular, guttate, and inverse psoriasis, among others [1].

It is a chronic disorder with a genetic predisposition combined with several intrinsic and extrinsic triggering factors such as metabolic syndrome, hypertension, stressful life events, smoking, alcohol consumption, infection, mechanical stress, and medication among others [2].

The underlying pathophysiology involves various classes of T cells and their interactions with dendritic cells and cells involved in innate immunity, including neutrophils and keratinocytes [3].

Psoriatic arthritis (PsA) is a painful and progressive, destructive seronegative inflammatory musculoskeletal disease that occurs most frequently in people with psoriasis [4].

Psoriasis has significant stigmatization and a negative impact on the patient's quality of life. Patients experience difficulties like maladaptive coping responses, problems in body image, self-esteem, and self-concept, and also have feelings of stigma, shame, and embarrassment regarding their appearance [5].

The prevalence rates of psoriasis showed a worldwide variation and have been reported to range between 0.09% and 5.1%, affecting approximately 120–180 million people [6].

The World Health Organization (WHO) recognized and reported psoriasis as a serious Non-communicable Disease (NCD). The report underscored that too many people in the world suffer needlessly from psoriasis due to incorrect or delayed diagnosis, inadequate treatment options, insufficient access to care, and because of social stigmatization. The report also recognizes the urgent need to pursue multilateral efforts to raise awareness regarding psoriasis and to fight stigmatization suffered by those with psoriasis [7].

Even though it occurs worldwide, most studies on the prevalence of psoriasis that are available in the literature are regional, mostly from the United States and Europe with limited data on skin color and developing countries [8].

In Africa, clinic-based investigations have demonstrated a wide variation in the prevalence of psoriasis between different countries, with higher rates seen in eastern than western Sub-Saharan Africa. Psoriasis has been reported to affect 1.9–3.5% of individuals in Kenya, Uganda, and Tanzania (Eastern Africa) vs. 0.025–0.9% in Ghana, Nigeria, Senegal, and Mali (Western Africa) [9].

A study done at Ayder referral hospital among patients attending a dermatology clinic, in Mekelle, north Ethiopia found the proportion of psoriasis to be 5.4% [10]. Another study focusing on describing the frequency and clinical characteristics of psoriasis patients in Tigray, Ethiopia found an average incidence of 183 cases/year [11]. In a study done to categorize the cutaneous disease observed in skin biopsies at ALERT in Addis Ababa, Ethiopia, observed a psoriasis skin biopsy rate of 2% [12].

There are notable differences in clinical presentations of psoriasis between light-skinned and black-skinned individuals. In addition to greater disease severity, there is an increased impact of psoriasis on QoL in non-white individuals compared with white individuals [10].

In Ethiopia, a study on the prevalence and factors associated with anxiety among patients with common skin diseases, the prevalence of anxiety among psoriasis patients was 27% [13]. In another study that looked into management practice, quality of life, and associated factors in psoriasis patients attending a dermatological center at ALERT, Ethiopia, it was reported that 104 (50.2%) patients experienced a small effect and only 4 (2.0%) patients had an extremely large effect on their QoL [14].

The prevalence of psoriasis has increased globally recently, but the paucity of studies limits our understanding of the true prevalence of psoriasis in the skin of color. Information on associated factors and the quality of life of psoriasis in Ethiopia as well as the Amhara region is limited.

To the best of our knowledge, there are no published studies that look into the proportion, factors associated with it, and quality of life of psoriasis patients in the study area by the electronic searching method. Therefore, this cross-sectional study is designed to assess the proportion, factors associated with it, and the quality of life of psoriasis patients among patients who attend the Dermatology clinic at Tibebe Ghion comprehensive specialized and Addis Alem primary hospitals, North West Ethiopia. This study was conducted for the This study was conducted for the requirements of thesis and dissertation and also available in the institutional repository of Bahir Dar university [15].

## 2. Methods and Materials

### 2.1. Study Design and Settings

An institution-based cross-sectional study design was conducted from June 8 to September 10, 2022 among patients who attend dermatology clinic at Tibebe Ghion Comprehensive Specialized and Addis Alem Primary Hospitals in Bahir Dar. Bahir Dar is the capital city of Amhara's regional state. The study was conducted from June 8 to September 10, 2022.

### 2.2. Operational Definitions

#### 2.2.1. Psoriasis

A skin lesion with the characteristic feature of well-demarcated, erythematous plaque with adherent silvery scale in typical locations over the scalp, elbow, knee, buttocks, or other areas of the body or diagnosed as psoriasis by a senior resident or dermatologist [1].

#### 2.2.2. Age of Disease Onset

<40 years (type 1 or early onset psoriasis) or >40 years (type 2 or late onset psoriasis) [1].

#### 2.2.3. BSA

Measurements of body surface involved by psoriatic lesions using a rule of nine and assuming one palm reflects approximately 1% of BSA. Mild psoriasis is defined as BSA of <10%, moderate psoriasis as 10–30%, and severe psoriasis as >30% [1].

#### 2.2.4. Stressful Life Event

Experience of any one or more of the following life events (death of close family, financial loss/problem, family conflict, major personal illness/injury, trouble at work environment, failure in exam, illness of family member, unemployment, or other) over the past one year [16].

#### 2.2.5. Alcohol Consumption

Individuals consuming >60 ml daily are excessive drinkers, Individuals consuming <60 ml once in a while and not regularly are occasional drinkers and abstainers are those who did not drink at all [17].

#### 2.2.6. Smoking

Heavy smokers are those who smoke >14 cigarettes per day for greater than 10 years or less than 10 years. Lighter smokers are those who smoke <14 cigarettes per day for less than 10 years [18].

#### 2.2.7. Hypertension

Blood pressure of 140/90 or more and/or current use of anti-hypertensive medications [19].

#### 2.2.8. Body Mass Index (BMI)

Expressed as kg/m^2^ (<18.5 kg/m^2^ = Underweight, 18.5–24.9 kg/m^2^ = normal weight, >25 kg/m^2^ = Overweight) [20].

#### 2.2.9. Dermatology Quality of Life Index (DLQI)

A validated (both English and Amharic versions) 10‐point questionnaire generates a score from zero to 30. It includes itch severity, embarrassment, shopping, clothing, social activity, sporting, work or study, relationship, sexual activity, and, the problem with treatment over the last week. Scores of 0-1 no effect at all on patient's life, 2–5 small effect on patient's life, 6–10 moderate effect on patient's life, 11–20 very large effect on patient's life and, 21–30 extremely large effect on patient's life [21].

### 2.3. Participants and Sampling

All patients attended the Dermatologic clinic at Tibebe Ghion comprehensive specialized and Addis Alem primary hospitals during the study period during data collection were included in the study whereas those with incomplete data during the data collection period were excluded from the study.

The sample size was calculated by using the formula for estimation of single population proportion. Based on the single population proportion assumptions were: A 95% confidence level (*Z*), 5% margin of error (*E*), and the prevalence of large negative effect of quality of life was 75% (from from a survey done by National Psoriasis Foundation, US) in the previous study [22]. The final sample size was 318 after adding 10% non-respondent.

Systematic random sampling was applied to select study participants from the Dermatologic clinic. The average number of patients who were admitted in Tibebe Ghion comprehensive specialized hospital and Addis Alem primary hospital during the data collection period was estimated based on the previous report, which was obtained by referring to a three-month registration book/record before data collection. Around 1660 patients were attended in Tibebe Ghion comprehensive specialized hospital and Addis Alem primary hospital dermatologic clinics in three months. The data were collected within a three-month duration. The sampling interval (Kth unit) was obtained by dividing the entire patients who attend the dermatologic clinic by the desired sample size (318) and it was approximately 5.22. The first patient was randomly chosen by the lottery method, and then every 5^th^ patient who attended the clinic was recruited for the study.

### 2.4. Data Collection Procedure

A structured and standardized interviewer-administered questionnaire was developed based on the objective of the study. A format that contains study variables of interest was prepared. An interviewer administered questionnaire (supplementary file (available here)) using the Dermatologic life quality index (DLQI) which is a validated tool to assess the impact of psoriasis on quality of life. Blood pressure measurement was measured using a manual sphygmomanometer or cuff. The weight of patients was measured using a digital weight scale. The height of patients was measured using a tape meter. BMI was calculated by dividing the weight of the patient by the height of square meters.

### 2.5. Data Management and Analysis

Data were coded and entered into EPI data version 3.1 and then exported to SPSS version 27 for analysis. Descriptive statistics and binary logistic regressions were computed. Binary logistic regression was used to select variables associated with psoriasis. In binary logistic regression, both bivariate and multivariable logistic regression were computed. In Bivariate analysis, independent variables with a *p* value <0.25 were selected as candidates for multiple logistic regressions. In multivariable logistic regression, statistically significant was considered at *p* < 0.05. AOR and their 95% confidence interval (CI) were used to measure the strength of the association.

### 2.6. Data Quality Assurance

The questionnaire for the interview was prepared in English and was translated into Amharic, which was re-checked by another translator for consistency in the meaning of words and to avoid misinterpretation. Four 3^rd^ year postgraduate students specializing in dermatovenereology collected the data. A pretest was done on 20 patients before the actual data collection started at Tibebe Ghion comprehensive specialized hospital. The principal investigator supervised the data collection. Each day the investigator checked the completeness and consistency of data collected by each collector. To keep the quality of data detailed training was given for data collectors and followed day-to-day activities during data collection.

### 2.7. Ethical Considerations

Ethical approval was obtained from the Institutional Review Board (IRB) of Bahir Dar University, College of Medicine and Health Sciences with reference number 508/2022. The IRB of Bahir Dar University decided and approved that verbal informed consent obtained from each study participant could be enough to be ethically assured of the research process. This was because unless the name and the participants' medical registration number (MRN) were used during data collection, there is no ethical issue that will be raised. An official letter was submitted to Tibebe Ghion Specialized and Addis Alem primary hospitals. Furthermore, all of the study participants were informed about the purpose of the study, and verbal consent was obtained. Issues related to confidentiality and any potential risk and benefits from participation in the study was discussed. In addition, participants were informed that participation is voluntary and that they can withdraw at any time without any precondition.

## 3. Results

### 3.1. Sociodemographic and Behavioral Characteristics of Study Participants

A total of 318 study participants were enrolled in this study with a response rate of 100%. The mean age of the study participants with a standard deviation (SD) was 22±18.2 years. Hundred seventy (53.5%) of the participants were males. About hundred eighty-eight (59.1%) were from urban areas. Ninety-six (30.2%) participants were married. Regarding educational status, 57 (19.7%) of the study participants cannot read and write and 19 (6.0%) had an educational status of higher education. Among the participants, 38 (11.9%) were farmers. 57 (17.9%) study participants had an average monthly family income of <860 ETB (Table 1).

### 3.2. Clinical Characteristics of Psoriasis Patients

Fourteen (82.4%) of psoriasis patients had onset of disease before 40 years of age. Thirteen (76.4%) of psoriasis patients had a disease duration of less than 5 years. One (5.9%) psoriasis patient had a positive family history. The majority of psoriasis patients reported a history of pruritus. The majority (88.2%) of psoriasis patients had plaque-type morphology. Most (82.3%) of them had multiple site involvement. Ten (58.8%) of psoriasis patients had body surface area involvement of 10–30% (Table 2).

### 3.3. Prevalence of Psoriasis

The prevalence of psoriasis was found to be 5.3% with 95% CI (3.1%, 8.4%).

### 3.4. Factors Associated with Psoriasis

Binary logistic regression was done to determine factors associated with psoriasis. First, all factors were analyzed by bivariate analysis, of them smoking, hypertension, alcohol drinkers, and stressful events were crudely associated with psoriasis at *p* < 0.05.

Multivariable logistic regression analysis was carried out using variables in the binary logistic regression with a *p* value <0.25 and stressful life events and alcohol consumption were significantly associated with psoriasis. Hence those who had stressful life events were about 3 times more likely to develop psoriasis than those who didn't. (AOD = 3.35 (1.13, 9.90) 95% CI *p* value of 0.03). Alcohol drinkers were about 4 times more likely to develop psoriasis than non-alcoholics (AOD = 3.73 (1.03, 13.51) 95% CI *p* value of 0.045) (Table 3).

### 3.5. Quality of Life of Psoriasis Patients

**Figure 1:**
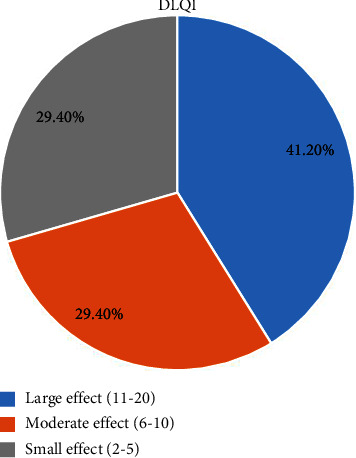
Quality of life of psoriasis patients who attend the dermatology clinic at TGSH and AAPH from June 8 to September 10, 2022.

Seven (41.2%) psoriasis patients had a large effect on their quality of life. Five (29.4%) had a moderate effect and another five (29.4%) patients had a small effect on their quality of life. The mean DLQI was 9.82 (Figure 1).

Regarding domains of dermatology quality of life index six (35%) psoriasis patients claimed the itch, soreness, pain, or stinging sensation was very much concerning to them (Table 4).

## 4. Discussion

This study found that the proportion of psoriasis was 5.3%. This is comparable with a study done at Ayder referral hospital, Mekelle, Ethiopia which was 5.4% [9]. This may be due to the setting and study design applied in both studies. The proportion is higher than the studies done in dermatology centers in India, Pakistan, Egypt, Nigeria, and Tanzania where the reported prevalence of psoriasis was 4.4%, 3.8%, 1.3%, 1.13%, 0.6%, and 0.09% respectively [23–26]. This difference might be because of the study design for example retrospective chart review was applied for 20 years in the Nigerian study. The proportion was higher than prevalence reports in the USA, Israel, Spain, and China which reported 3.2, 3.2. 1.7%, and 0.47% respectively [26–29]. The difference might be due to study design as these were population-based studies.

The prevalence was lower compared to prevalence reports from Denmark, Malaysia, and Norway where they reported prevalence of 7.9%, 9.5%, and 11.43% respectively [7, 30]. The difference is due to setting and study design as the Denmark study is the lifetime prevalence of psoriasis and the Norway study also includes patients with both physician-diagnosed and self-diagnosed psoriasis.

The finding is compatible with worldwide and international prevalence studies which have been reported to range from 0.09% to 5.1% [31]. This is due to worldwide variation of psoriasis occurrence as genetic background and environment are key factors in determining psoriasis.

This study found 14 (82.4%) of psoriasis patients had type 1 or disease onset before 40 years. This is similar to a study done at Ayder referral hospital, Mekelle, Ethiopia where more than two third (71%) of patients had onset before 40 years of age, and international studies where the majority of psoriasis patients have type 1 psoriasis [1].

This study found a male-to-female ratio of 1.8 : 1 which is similar to the ratio in Ayder's study where the male-to-female ratio was 1.6 : 1 [9] which is in comparison to a study done in Egypt that reported that 52% of cases were females [32]. The difference in prevalence based on gender is lacking even in international studies currently and needs further study.

This study found a positive family history of 1 (5.9%). This is comparable with a study done at Ayder where positive family history was found at 9.5% [33].

This study found alcohol consumption was significantly associated with psoriasis. This is similar to a finding of a systematic review of literature which concluded that alcohol consumption was significantly associated with psoriasis. However, it remains still unclear whether alcohol represents a genuine risk factor or is merely a consequence of psoriasis as only four studies concluded that alcohol was a risk factor for psoriasis [33].

This study found that stressful life events were significantly associated with psoriasis, this is similar to a finding in a systematic literature review and meta-analysis which found a significant association between stressful life events and psoriasis. However, it remains unclear whether stressful life events are a genuine risk factor for the onset of psoriasis as only five case-control studies evaluating stressful events preceding the onset of psoriasis [34].

This study found seven (41.2%) of psoriasis patients had a large negative effect on their quality of life and the mean dermatology quality of life index (DLQI) was 9.82. This is higher than a study done in the ALERT center where the majority (50.2%) of patients experienced a small effect and only 13% of patients had a large effect on their quality of life with a mean DLQI of 6.25 [14]. This might be due to differences in study design and a large number sample size employed in the ALERT center.

The finding is comparable with that of a survey done by the National Psoriasis Foundation where nearly 75% of patients had a moderate to the large negative impact on their quality of life (QoL) [22]. In contrast, this finding was lower than that of a study conducted at a referal hospital in Northwest Ethiopia [35] where the mean dermatology quality of life index (DLQI) score was 13.05. This might be due to a difference in sample size since this study enrolled a lower number of participants, which may result in such variations.

### 4.1. Strengths and Limitations of the Study

This study assessed the quality of life of psoriasis patients descriptively. This study was conducted at two dermatology centers. Data was collected by senior residents with a consultation with dermatologists. Although this study may serve as informative on disease burden it didn't allow generalization at a population level as it is done in a hospital setting. The study design was cross-sectional where true causal inference between associated factors and psoriasis cannot be drawn. Prospective and retrospective follow-up of patients based on their exposure or outcome status would be appropriate to detect casual relationships. Future studies should answer whether stressful life events and alcohol are responsible for the onset or sole triggers of psoriasis using case-control and cohort studies.

## 5. Conclusions

The proportion of psoriasis in this study was comparable with international studies and previous studies were in Ethiopia but substantially higher than studies from other African countries. Stressful life events and alcohol consumption were significantly associated with psoriasis. Whether they are responsible for disease onset or the sole aggravating factor needs further investigation. Addressing the psychosocial component of the disease and appropriate screening for alcohol usage and a strategy to limit intake should be discussed with patients. The majority of psoriasis patients had a large negative effect on their quality of life. Assessing the quality-of-life impairment caused by psoriasis should be an integral part of planning therapy.

## Figures and Tables

**Table 1:** Sociodemographic and behavioral characteristics of patients who attend the dermatology clinic at TGSH and AAPH from June 8 to September 10, 2022.

Sociodemography	Category	Frequency	Percentage (%)
Age group	<40	268	84.3
	>40	50	15.7

Sex	Male	170	53.5
	Female	148	46.5

Residence	Rural	130	40.9
	Urban	188	59.1

Marital status	Single	149	46.9
	Married	96	30.2
	Divorced	2	0.6
	Widowed	3	0.9

Educational status	Cannot read and write	57	17.9
	Elementary	106	33.3
	Secondary	92	28.9
	Higher	19	6.0

Occupation	Student	93	29.2
	Farmer	38	11.9
	Merchant	34	10.7
	Government employee	18	5.7
	Housewife	43	13.5

Income^∗^	Very low (<860)	57	17.9
	Low (861–1500)	61	19.2
	Average (1501–3000)	58	18.2
	Above average (3001–5000)	100	31.4
	High (>5000)	42	13.2

Smoking	Yes	20	10
	No	180	90

Alcohol	Yes	53	26.5
	No	147	73.5

Stress	Yes	58	40.8
	No	142	59.2

BMI	<18.5	4	1.9
	18.5–24.9	196	93.8
	>25	9	4.3

HTN	Yes	15	7.5
	No	185	92.5

^∗^Based on the Ethiopian civil service monthly salary scale for civil servants.

**Table 2:** Clinical characteristics of psoriasis patients who attend the dermatology clinic at TGSH and AAPH from June 8 to September 10, 2022

Clinical feature	Category	Frequency	Percentage
Family history of psoriasis	Yes	1	5.9
	No	16	94.1

Age at onset	<40	14	82.4%
	>40	3	17.6%

Disease duration	<5	13	76.4%
	>5	4	23.6%

Symptom	Pruritus	16	94%
	Burning	1	6%
	Joint pain	0	0

Morphology	Plaque	15	88.2%
	Sebopsoriasis	1	6.2%
	Pustular	1	6.2%

Body site	Multiple sites	14	82.3%
	One site	3	17.7%

BSA involvement	<10	7	41.2%
	10–30	10	58.8%
	>30	0	0%

BSA: Body Surface Area.

**Table 3:** Factors associated with psoriasis among patients who attend the dermatology clinic at TGSH and AAPH from June 8 to September 10, 2022

Variable	Category	Psoriasis		COR (95%)	AOD (95%)	*P* value
		Yes	No			
Marital status	Single	8	141	1	1	
	Married	9	92	1.72 (1.94, 2.05)	1.10 (3.05, 7.28)	0.948

Smoking	Yes	7	13	11.35 (4.67, 19.55)	3.60(4.06, 12.01)	0.532
	No	10	187	1	1	

Alcohol	Yes	9	44	3.55 (1.29, 9.76)	3.73(1.03, 13.51)	0.045^∗^
	No	8	139	1	1	

Stress	Yes	12	46	3.07 (1.12, 8.42)	3.35 (1.13, 9.90)	0.028^∗^
	No	5	137	1	1	

HTN	Yes	7	8	15.06(17.90, 25.2)	4.61 (5.19, 10.9)	0.344
	No	10	234	1	1	

^∗^Significantly associated *p* value <0.05, HTN: Hypertension.

**Table 4:** Dermatology quality of life index domains of psoriasis patients among patients attending the dermatology clinic at TGSH and AAPH from June 8 to September 10, 2022.

Domain	Very much	A lot	A little	Not at all	Not relevant
How itchy, sore, painful or stinging has your skin been?	6 (35%)	5 (30%)	6 (35%)	0	0
How embarrassed or self-conscious have you been because of your skin?	2 (11.7%)	6 (35%)	6 (35%)	3 (17.6%)	0
How much has your skin interfered with you going shopping or looking after your home or garden?	2 (11.7%)	8 (47%)	4 (23.5%)	3 (17.6%)	0
How much has your skin influenced the clothes you wear?	2 (11.7%)	4 (23.5%)	6 (35%)	5 (30%)	0
How much has your skin affected any social or leisure activities?	2 (11.7%)	5 (30%)	1 (5.8%)	9 (52.9%)	0
How much has your skin made it difficult for you to do any sport?	0	0	6 (35%)	7 (41%)	9 (52.9%)
Has your skin prevented you from working or studying?	0	7 (41%)	6 (35%)	4 (23.5%)	0
How much has your skin created problems with your partner or any of your close friends or relatives?	2 (11.7%)	0	8 (47%)	7 (41%)	0
How much has your skin caused any sexual difficulties?	0	2 (11.7%)	3 (17.6%)	5 (30%)	7 (41%)
How much of a problem has the treatment for your skin been, for example by making your home messy, or by taking up time?	2 (11.7%)	2 (11.7%)	3 (17.6%)	10 (58.8%)	0

## Data Availability

All data relevant to the study are included in the article or uploaded as supplementary.

## Abbreviations

AAPH: Addis Alem primary hospital
ALERT: All African leprosy rehabilitation and training center
ARHB: Amhara regional health bureau
BoD: Burden of disease
BMI: Body mass index
BSA: Body surface area
CASPAR: Classification criteria for psoriatic arthritis
CI: Confidence interval
CVD: Cardiovascular disease
DQLI: Dermatology quality of life index
GBD: Global burden of disease
HRQoL: Health-related quality of life
IPC: International psoriasis council
MoH: Ministry of health
MTX: Methotrexate
NCD: Non-communicable diseases
PASI: Psoriasis area severity index
PsA: Psoriatic arthritis
PUVA: Psoralen and ultraviolet A
QoL: Quality of life
RR: Relative risk
T2DM: Type 2 diabetes mellitus
TGSH: Tibebe Ghion specialized hospital
URTI: Upper respiratory tract infection
USA: United States of America
UV: Ultraviolet
WHO: World Health Organization.

## Supplementary Materials

Supplementary Materials Supplementary File: Questionnaire.
