# Supplementary material for: Magnitude of Psoriasis and Its Associated Factors, and Quality of Life of Psoriasis Patients among Patients Who Attend Dermatology Clinic at Tibebe Ghion Comprehensive Specialized and Addis Alem Primary Hospitals, North West Ethiopia, 2022: Institution-Based Cross-Sectional Study
Source: Dermatol Res Pract. 2024 Sep 18;2024:5560174. doi: 10.1155/2024/5560174 (PMC11424841; doi:10.1155/2024/5560174)
Supplement: Supplementary Materials — Supplementary File: Questionnaire. [file 5560174.f1.pdf]

**Questionnaire**

## 1.Sociodemography

Age ___

Sex
- Male☒
- Female ☐

Place of residence
- Rural ☐
- Urban ☐

Education
- Can't read &write ☐
- Primary ☐
- Secondary ☐
- Higher ☐

Marital status
- Single☐
- Married☐
- Divorced☐
- Widowed☐

Occupation
- Student ☐
- Farmer ☐
- Merchant ☐
- Government employee ☐
- Housewife ☐

Average monthly income
- <860 ☐
- 861-1500 ☐
- 1500-3000 ☐
- 3001-5000 ☐
- >5001 ☐

## 2.Clinical feature

Age at onset
- <40 years of age ☐
- >40 years of age ☐

Disease duration
- <5 years ☐
- >5 years ☐

Family history
- Yes ☐
- No ☐

Symptom
- Pruritus ☐
- Burning ☐
- Joint pain ☐
- Other_____

Morphology
- Plague type ☐
- Guttate ☐
- Erythrodermic ☐
- Pustular ☐
- Sebopsoriasis ☐
- Other ________

Site
- Multiple ☐
- Single ☐

Extent
- <10% ☐
- 10-30% ☐
- >30% ☐

## 3.Factors

Alcohol use
- Yes ☐
  - Excessive ☐
  - Occasional ☐
  - Can't quantify ☐
- No ☐

Stressful life event
- Yes ☐
  - Death of close family ☐
  - Financial loss ☐
  - Family conflict ☐
  - Major personal illness ☐
  - Change in work conditions ☐
  - Failure in examination ☐
  - Illness of family member ☐
  - Miscellaneous ☐
- No ☐

Smoking
- Yes ☐
  - Heavy ☐
  - Lighter ☐
- No ☐

Hypertension
- Yes ☐
- No ☐

BMI
- <18.5 ☐
- 18.5-24.9 ☐
- >25 ☐

## 4. Dermatology Life Quality Index (DQLI): English Version

This questionnaire aims to measure how much your skin problem has affected your life OVER THE LAST WEEK. Please tick (✓) one box for each question.

**Total** ☐

| | | | | |
|---|---|---|---|---|
| 1 | Over the last week, how itchy, sore, painful, or stinging has your skin been? | Very much<br>A lot<br>A little<br>Not at all | ☐<br>☐<br>☐<br>☐ | |
| 2 | Over the last week, how embarrassed or self-conscious have you been because of your skin? | Very much<br>A lot<br>A little<br>Not at all | ☐<br>☐<br>☐<br>☐ | |
| 3 | Over the last week, how much has your skin interfered with you going shopping or looking after your home or garden? | Very much<br>A lot<br>A little<br>Not at all | ☐<br>☐<br>☐<br>☐ | Not relevant☐ |
| 4 | Over the last week, how much has your skin influenced the clothes you wear? | Very much<br>A lot<br>A little<br>Not at all | ☐<br>☐<br>☐<br>☐ | Not relevant☐ |
| 5 | Over the last week, how much has your skin affected any social or leisure activities? | Very much<br>A lot<br>A little<br>Not at all | ☐<br>☐<br>☐<br>☐ | Not relevant☐ |
| 6 | Over the last week, how much has your skin made it difficult for you to do any sport? | Very much<br>A lot<br>A little<br>Not at all | ☐<br>☐<br>☐<br>☐ | Not relevant☐ |
| 7 | Over the last week, has your skin prevented you from working or studying? | Yes<br>No | ☐<br>☐ | Not relevant☐ |
| | If "No", over the last week how much has your skin been a problem at work or studying? | A lot<br>A little<br>Not at all | ☐<br>☐<br>☐ | |
| 8 | Over the last week, how much has your skin created problems with your partner or any of your close friends or relatives? | Very much<br>A lot<br>A little<br>Not at all | ☐<br>☐<br>☐<br>☐ | Not relevant☐ |
| 9 | Over the last week, how much has your skin caused any sexual difficulties? | Very much<br>A lot<br>A little<br>Not at all | ☐<br>☐<br>☐<br>☐ | Not relevant☐ |
| 10 | Over the last week, how much of a problem has the treatment for your skin been, for example by making your home messy, or by taking up time? | Very much<br>A lot<br>A little<br>Not at all | ☐<br>☐<br>☐<br>☐ | Not relevant☐ |

Please check you have answered EVERY question. Thank you
